# Supplementary material for: CircRNAs Related to Breast Muscle Development and Their Interaction Regulatory Network in Gushi Chicken
Source: Genes (Basel). 2022 Oct 29;13(11):1974. doi: 10.3390/genes13111974 (PMC9689937; doi:10.3390/genes13111974)
Supplement: Supplementary file 1 [file genes-13-01974-s001.zip › Table S1.pdf]

Table S1. Primers list.

Target	Primer sequence (5'-3')	Product length (bp)	AT(°C)
gga_circ_0000345	F：GCCAGCACTGATATGGACAA	218	55
	R：TCCCTTCCTTTGCAATCCAG		
gga_circ_0004580	F：GATTGGAGTGCGTCAAGTCC	147	57
	R：TGCCAATGATTGGCTGCTGT		
gga_circ_0005943	F：TTGAGACGAGCTGTCCCTCA	139	57
	R：GTCTCAGGGCAATCAAGGCA		
gga_circ_0006078	F：TGAGGCCAGCCTGGCACA	178	59
	R：CACTGGCATTGCACAGGAAC		
gga_circ_0006568	F：CATGGTGGGAGAAGCCTTAG	182	57
	R：GTCATGGATTCCTTGACGAGC		
gga_circ_0006619	F：GGTCCCAAAGGACAAGTTGT	173	57
	R：CGGTCTGTAGCCAGCATTAC		
gga_circ_0006876	F：AGAAGTGCTTTGCAGCTTGC	181	55
	R：AGAACAGAAATGTGCTGGCG		
gga_circ_0008159	F：GCGATACCAACAGAACCGTC	154	57
	R：TGCTCCGTCATAGCGGGATA		
β-actin	F：CACGGTATTGTCACCAACTG	200	57
	R：ACAGCCTGGATGGCTACATA		

Abbreviation: AT refers to the annealing temperature; F and R refer to the forward and reverse primers, respectively.
