# Supplementary material for: CircRNAs Related to Breast Muscle Development and Their Interaction Regulatory Network in Gushi Chicken
Source: Genes (Basel). 2022 Oct 29;13(11):1974. doi: 10.3390/genes13111974 (PMC9689937; doi:10.3390/genes13111974)
Supplement: Supplementary file 1 [file genes-13-01974-s001.zip › Table S2.pdf]

Table S2. Sequencing output and quality evaluation statistics of 12 libraries.

Sample	Raw_reads	Clean_reads	Clean_bases	Error_rate(%)	Q20(%)	Q30(%)	GC_content(%)
W6_1	117,197,064	111075916	16.66 G	0.01	97.55	93.68	50.24
W6_2	98,255,520	93593136	14.04 G	0.01	97.70	94.00	50.72
W6_3	89,496,872	84971878	12.75 G	0.01	97.63	93.83	50.86
W14_1	101,944,936	96542790	14.48 G	0.02	97.20	92.88	53.12
W14_2	96,278,608	91219510	13.68 G	0.01	97.48	93.57	53.00
W14_3	114,606,992	108973446	16.35 G	0.01	97.60	93.77	52.03
W22_1	106,845,354	103147962	15.47 G	0.02	97.20	92.88	53.87
W22_2	89,750,270	85885112	12.88 G	0.02	96.48	91.06	51.51
W22_3	96,537,600	92626188	13.89 G	0.02	96.90	92.05	51.54
W30_1	105,872,366	101782844	15.27 G	0.01	97.79	94.25	50.53
W30_2	98,889,740	94051690	14.11 G	0.01	97.56	93.69	51.85
W30_3	100,815,554	95671700	14.35 G	0.01	97.65	93.86	52.30

Abbreviations: W6_1, sample 1 of 6 weeks; W6_2, sample 2 of 6 weeks; W6_3, sample 3 of 6 weeks; W14_1, sample 1 of 14 weeks; W14_2, sample 2 of 14 weeks; W14_3, sample 3 of 14 weeks; W22_1, sample 1 of 22 weeks; W22_2, sample 2 of 22 weeks; W22_3, sample 3 of 22 weeks; W30_1, sample 1 of 30 weeks; W30_2, sample 2 of 30 weeks; W30_3, sample 3 of 30 weeks.
