# Supplementary figures and images for: CircRNAs Related to Breast Muscle Development and Their Interaction Regulatory Network in Gushi Chicken
Source: Genes (Basel). 2022 Oct 29;13(11):1974. doi: 10.3390/genes13111974 (PMC9689937; doi:10.3390/genes13111974)

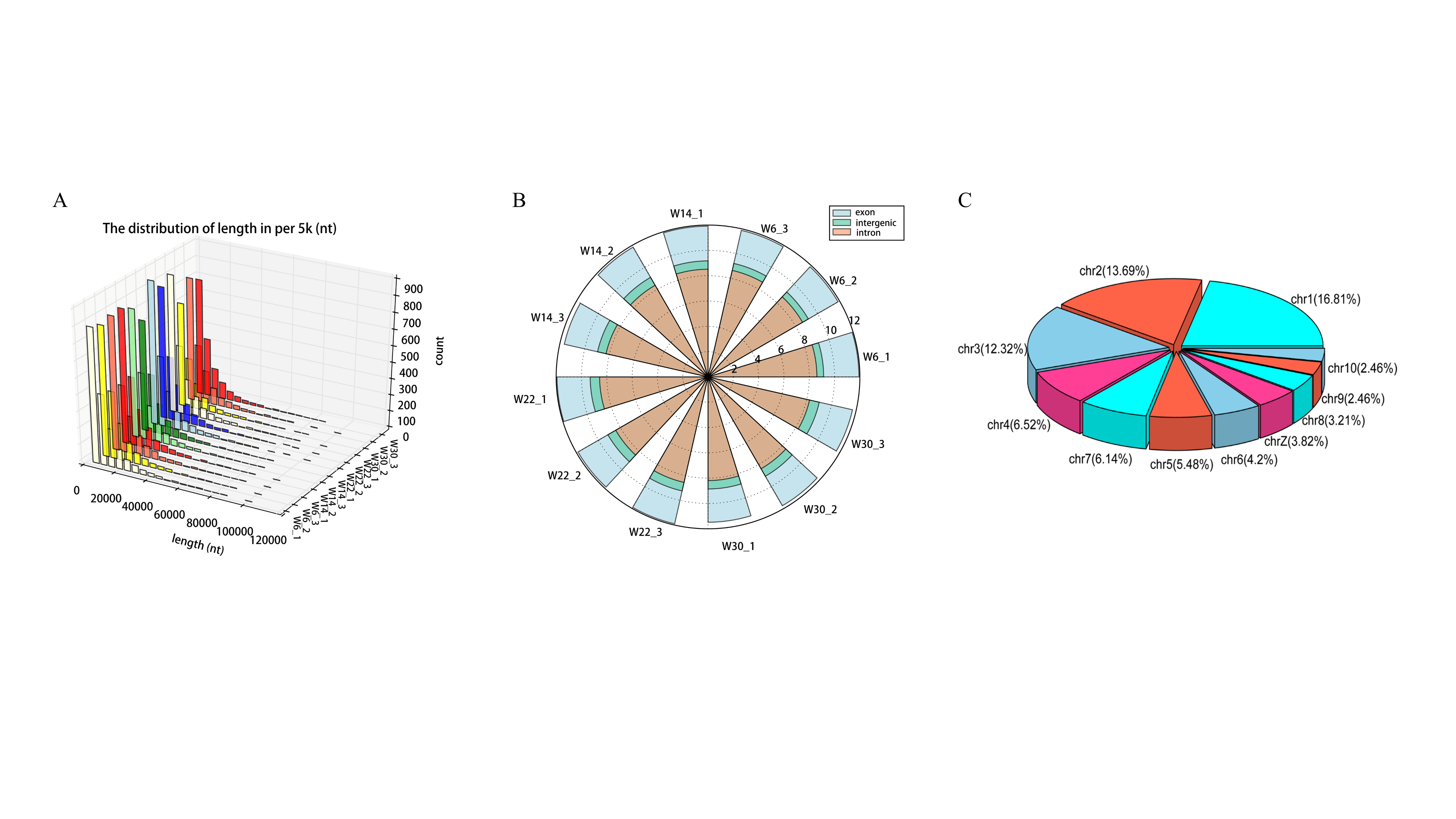

Supplement: Supplementary file 1 [file genes-13-01974-s001.zip › Supplementary Figure S1.tif]

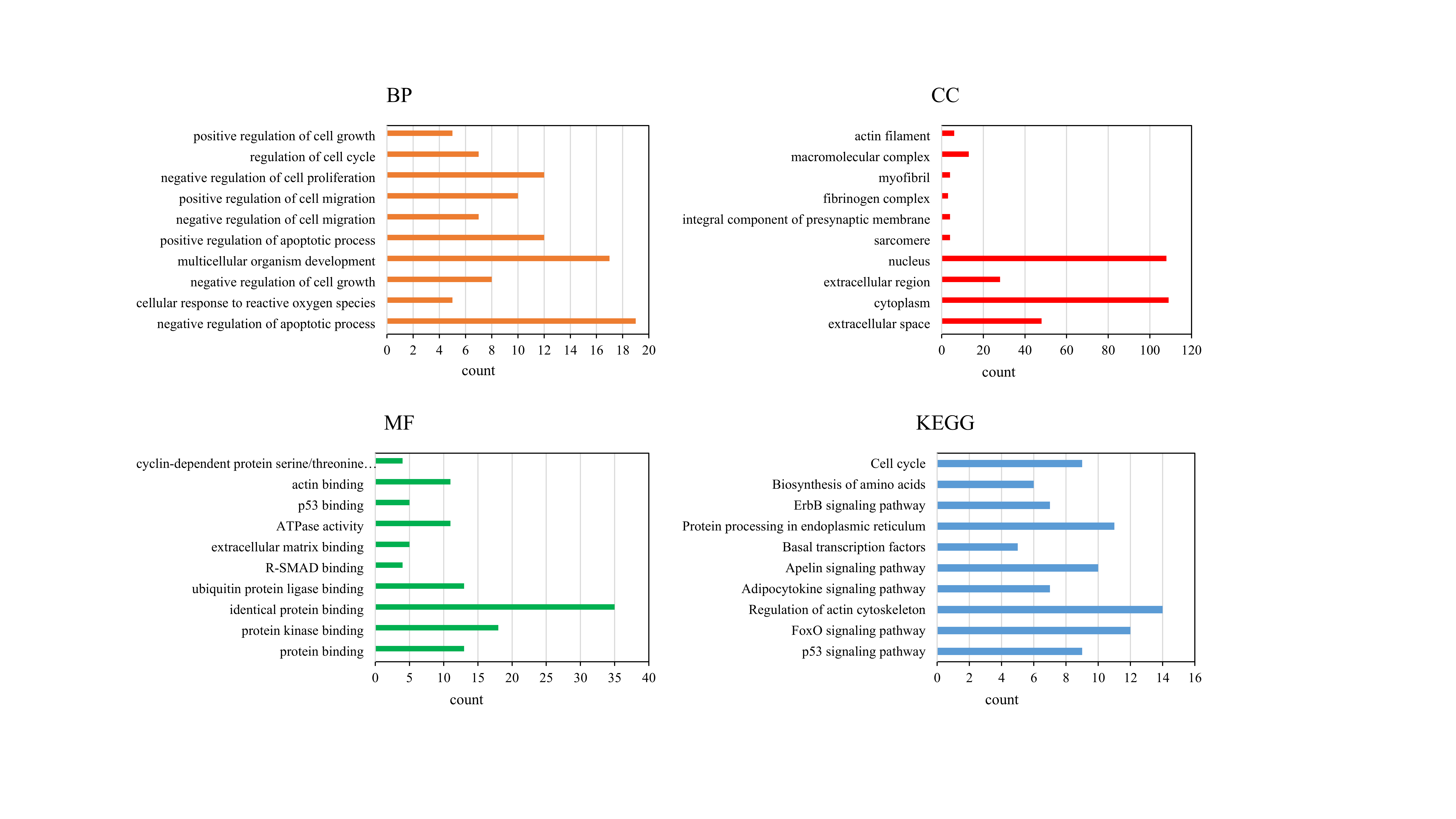

Supplement: Supplementary file 1 [file genes-13-01974-s001.zip › Supplementary Figure S2.tif]

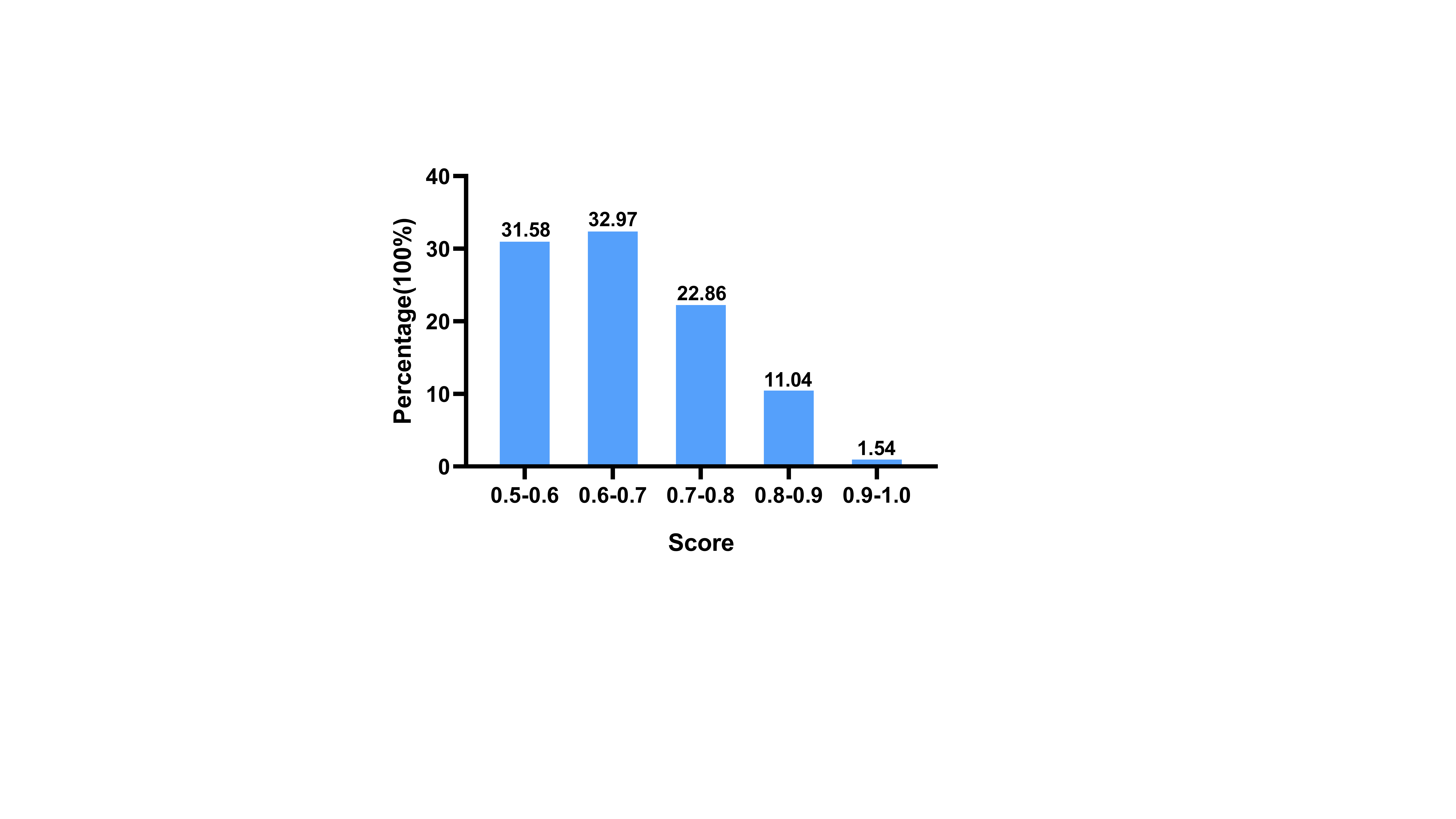

Supplement: Supplementary file 1 [file genes-13-01974-s001.zip › Supplementary Figure S3.tif]
